# 

**Published:** 1894-12-08

**Authors:** 


The Divorce Between Medicine and Surgery - 163

Hypertrophy of the Prostrate - 164

The Study of Man. IX.—Language and Race in Europe - 165

The First Century of English Medical Literature. X. The Library of the Good Huswife - 166

Notes and News - 168

Medical Progress and Hospital Clinics:

Cystitis - 169

Traumatic Ulnar Nerve Paralysis.—II. - 170

PROGRESS IN SURGERY.—Surgery of the Vermiform Appendix - 171

PROGRESS IN MEDICINE.—Diseases of the Nervous System - 173

NEW DRUGS AND APPLIANCES - 174

Annotations.—How Sanitation is Done in Ireland—Rolling of the Head—Compulsory Removal to Hospitals - 167

The Institutional Workshop:

WITHIN THE HOSPITALS.—All Saints Convalescent Hospital, Eastbourne - 175

THE STORY OF THE INSANE FROM YEAR TO YEAR - 176

THE MIDDLESEX HOSPITAL - 177

PRACTICAL DEPARTMENTS.—Bedsteads - 177

CONSTRUCTION NOTES - 178

Scraps and Gleanings - 178

The Book World of Medicine and Science - 179

The Student of Medicine - 179

The Government Civil Hospital, Hong Kong - lxvi

Old Time Nurses. V.—Out of Work - lxvii

Dress and Uniforms - lxviii

Royal British Nurses' Association - lxix

From Two to Three Guineas - lxix

Notes from India - lxix

Notes and Queries - lxix

Everybody's Opinion - lxx

Presentations—Death in Our Ranks - lxx

